# Disseminated diffuse midline gliomas, H3K27-altered mimicking diffuse leptomeningeal glioneuronal tumors: a diagnostical challenge!

**DOI:** 10.1186/s40478-022-01419-3

**Published:** 2022-08-19

**Authors:** Arnault Tauziède-Espariat, Aurore Siegfried, Emmanuelle Uro-Coste, Yvan Nicaise, David Castel, Annick Sevely, Marion Gambart, Sergio Boetto, Lauren Hasty, Alice Métais, Fabrice Chrétien, Joseph Benzakoun, Stéphanie Puget, Jacques Grill, Volodia Dangouloff-Ros, Nathalie Boddaert, Azadeh Ebrahimi, Pascale Varlet, the RENOCLIP-LOC Network

**Affiliations:** 1 Department of Neuropathology, GHU Paris Psychiatrie Neurosciences, Sainte-Anne Hospital, 1, rue Cabanis, 75014 Paris, France; 2 Paris University France, 75006 Paris, France; 3 Department of Pathology, Toulouse University Hospital, 31300 Toulouse, France; 4 Cancer Research Center of Toulouse (CRCT), INSERM U1037, Toulouse, France; 5 U981, Molecular Predictors and New Targets in Oncology, INSERM, Gustave Roussy, Université Paris-Saclay, 94805 Villejuif, France; 6 Univ. Evry, Université Paris-Saclay, 91000 Evry, France; 7 Department of Radiology, Purpan University Hospital, 31300 Toulouse, France; 8 Department of Pediatric Oncology, Toulouse University Hospital, 31300 Toulouse, France; 9 Department of Neurosurgery, Toulouse University Hospital, 31300 Toulouse, France; 10 Department of Radiology, GHU Paris-Psychiatrie et Neurosciences, Sainte-Anne Hospital, Paris, France; 11 Department of Pediatric Neurosurgery, Necker Hospital, APHP, Université Paris Descartes, Sorbonne Paris Cite, 75015 Paris, France; 12 Department of Neurosurgery, CHU de Fort de France, Université Des Antilles, Paris, France; 13 Department of Pediatric Oncology, Gustave Roussy, Université Paris-Saclay, 94805 Villejuif, France; 14 Pediatric Radiology Department, Hôpital Necker Enfants Malades, AP-HP, Institut Imagine INSERM U1163 and U1299, 75015, Université Paris Cité, Paris, France; 15 Institute of Neuropathology, University of Bonn, Venusberg Campus 1, 53127 Bonn, Germany

Diffuse midline gliomas (DMG) are divided into four subtypes depending on their molecular characteristics, and/or location: DMG, H3.3 K27-mutant; DMG, H3.1 or H3.2 K27—mutant; DMG, H3-wildtype, with EZHIP overexpression and DMG, *EGFR*-altered [1]. Leptomeningeal dissemination at diagnosis has been variably reported depending on the series (up to 42%) [2]. Very little genetic and epigenetic data is available for those disseminated cases, with one case harboring a concomitant *FGFR1* mutation [3] and another a 1p deletion [4]. Consequently, their relationship with diffuse leptomeningeal glioneuronal tumors (DLGNT), remains unclarified.

Herein, we describe the histopathological, neuroradiological and molecular (including DNA-methylation profiling) features of three initially disseminated H3K27-altered tumors with glioneuronal features including two cases with an associated MAPK pathway alteration.

The cases concerned three females, aged 14, 13 and 40-year-old (see Additional file 1: Table S1). At the initial diagnosis, in all cases, the tumors were disseminated with supra-tentorial and infra-tentorial leptomeningeal infiltration. An intraparenchymal mono-thalamic involvement was observed in cases 1 and 2; case 3 did not present any intraparenchymal involvement, until the end of the follow-up (Fig. 1). A leptomeningeal biopsy was performed in all cases. Histopathologically, all tumors presented a glioneuronal immunophenotype, and, one of them also had numerous microcalcifications (Fig. 2 and Additional file 2: Table S2). A 1p deletion was evidenced in case 1 and therefore a diagnosis of DLGNT was suggested (Fig. 2D). NGS sequencing showed a *FGFR1* N546K mutation (case 1), a *BRAF* V600E mutation (case 2) and a *H3F3A* K27M mutation (case 3). The DNA-methylation profiling classified cases 1 and 3 as DMG, H3K27-altered, subtype H3K27M/EZHIP overexpressing (calibrated scores 0.99 and 0.82 respectively) and case 2 as DMG H27K27-altered, subtype *EGFR*-altered (calibrated score 0.95) (Additional file 3: Fig. S1). Complementary analyses found a loss of H3K27me3 (in all cases), an EZHIP overexpression (cases 1 and 2) (Fig. 2), but no *EGFR* alteration (all exons were tested by whole exome sequencing, and an amplification was ruled out by FISH analyses) was evidenced. Case 1 received several lines of chemotherapy and craniospinal radiation therapy but passed away 16 months after the initial diagnosis, whereas the case 2, treated by chemotherapy and targeted anti-BRAF therapy, is still alive with a stable disease, 7 months after the initial diagnosis. The patient 3 received chemotherapy and craniospinal irradiation but died 4 months after the diagnosis.

**Fig. 1:**
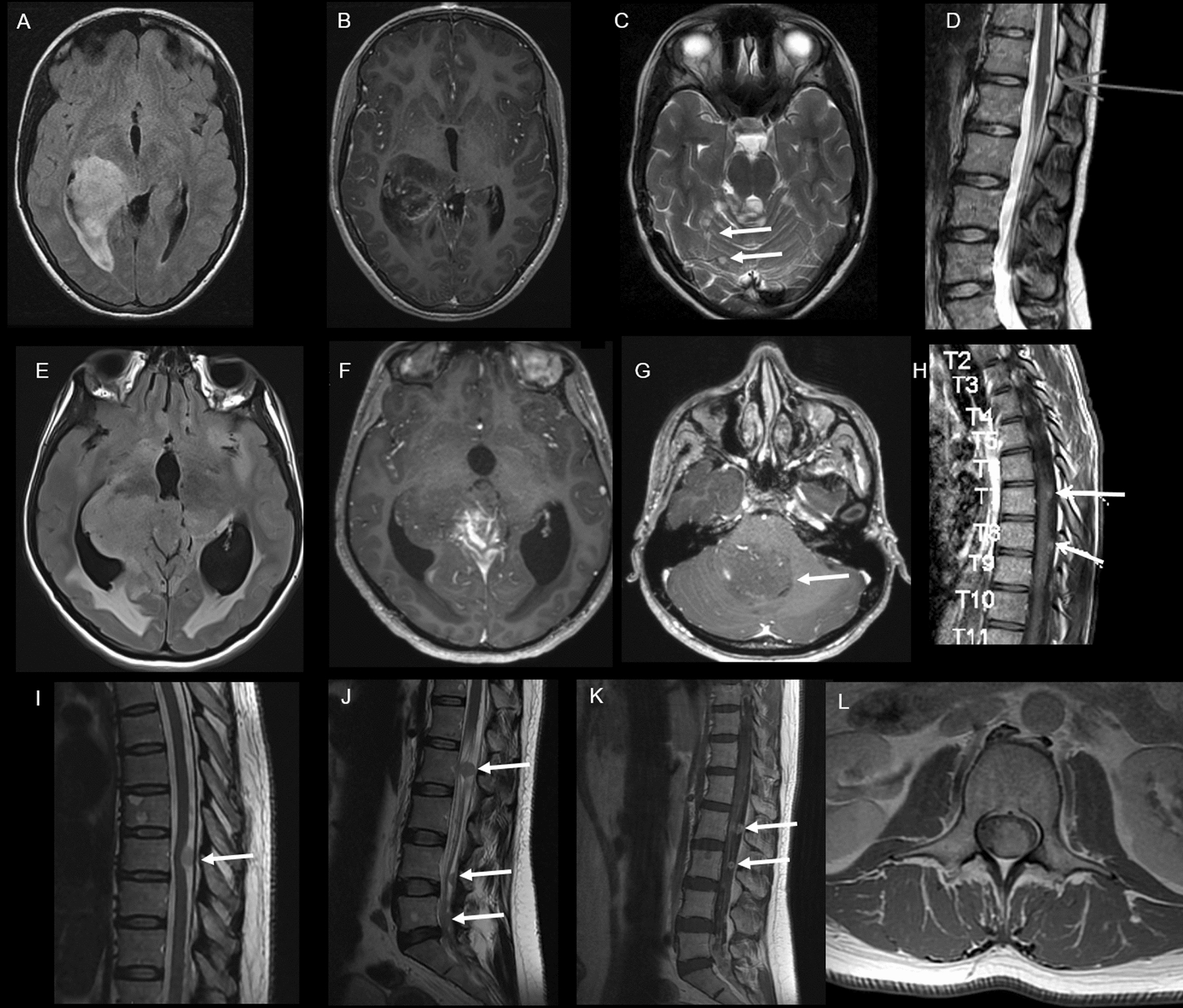
Radiological features. Case #1 **A** Axial FLAIR brain MRI shows a hyperintense infiltrative lesion of the right thalamus extended to the right lateral ventricle. **B** Axial contrast-enhanced T1-weighted brain MRI shows a heterogeneous enhancement after gadolinium injection. **C** Axial T2-weighted brain MRI shows other nodular FLAIR hyperintensities of the cerebellum (arrows). **D** Sagittal T2-weighted spine MRI shows a hyperintense peripheral lesion of the spinal cord (arrow). Case #2 **E** Axial FLAIR-weighted brain MRI shows a hyperintense lesion of the right thalamus extended to the third ventricle and the right hippocampus. **F** Axial contrast-enhanced T1-weighted brain MRI shows a heterogeneous enhancement of this lesion. **G** Axial contrast-enhanced T1-weighted brain MRI shows an intraventricular localization in the fourth ventricle (arrow). **H** Sagittal contrast-enhanced T1-weighted spine MRI shows multiple spinal leptomeningeal lesions. Case #3 **I** Sagittal T2-weighted spine MRI shows a thoracic hyperintense leptomeningeal lesion. **J** Sagittal T2-weighted lumbar MRI shows multiple lumbar intradural lesions, attached to nerve roots and in the lower end of the dural sac. **K** Sagittal and **L** Axial contrast-enhanced T1-weighted lumbar MRI show an enhancement of these lesions. FLAIR: Fluid Attenuated Inversion Recovery

**Fig. 2:**
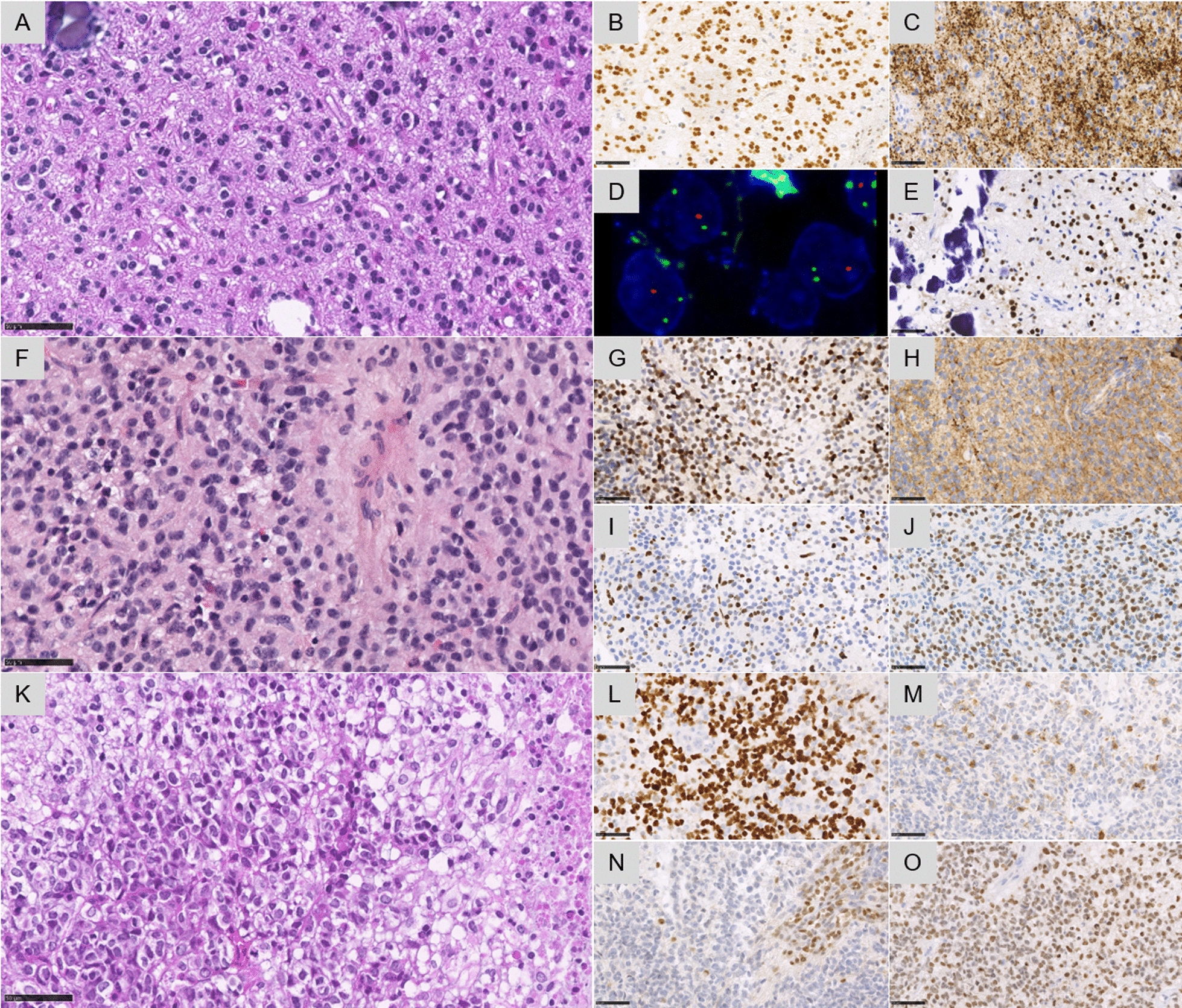
Histopathological and molecular features. Case #1 **A** A glial proliferation with oligo-like features and one microcalcification (HPS, magnification × 400). **B** Diffuse expression of Olig2 (magnification × 400). **C** Diffuse synaptophysin immunoreactivity without true neuropil islands (magnification × 400). **D** 1p deletion by FISH analysis (green signal for 1q25 and orange signal for 1p36, magnification × 400). **E** EZHIP overexpression in all tumor cells (magnification × 400). Case #2 **F** A glial proliferation with astrocytic features (magnification × 400). **G** Diffuse expression of Olig2 (magnification × 400). **H** Diffuse synaptophysin immunoreactivity without true neuropil islands (magnification × 400). **I** Loss of the trimethylation H3K27me3 in tumor cells (magnification × 400). **J** EZHIP overexpression in all tumor cells (magnification × 400). Case #3 **K** A high-grade glial proliferation with several mitoses and necrosis (magnification × 400). **L** Immunoreactivity for neurofilament in a subset of tumor cells (magnification × 400). **N** Loss of the trimethylation H3K27me3 in tumor cells (magnification × 400). **O** H3K27M immunopositivity in all tumor cells (magnification × 400). Black scale bars represent 50 μm

DLGNTs are glioneuronal tumors molecularly defined by a chromosome arm 1p deletion and a MAPK pathway alterations [1]. Contrary to what their name suggest, they can present a parenchymal component, which can include a thalamic location, with or without leptomeningeal involvement [5]. The already published H3K27M-mutant cases with a disseminated radiological presentation (including a case with a 1p deletion) raises the question of a potential overlap between DLGNT and DMG [3, 4, 6]. However, those cases did not have DNA-methylation analysis, and their relationship to DMG, H3 K27–altered remains open in the last version of the World Health Organization classification [1]. Herein, we present three initially disseminated leptomeningeal tumors, including one case with a 1p deletion and two with *BRAF/FGFR1* mutations, classified as DMG using DNA-methylation profiling. Like patients with DMG-H3K27 mutant with concomitant *BRAF* or *FGFR1* mutation, the two current disseminated cases H3K27-altered (one with EZHIP overexpression) with a MAPK mutation were older than classical DMG and histologically presented a glioneuronal immunophenotype and /or microcalcifications [7, 8]. The case 2, classified as DMG, H3K27-altered (*EGFR*-mutant) proven by DNA-methylation analysis, represents the first example of a disseminated presentation of this typically bithalamic tumor type [9]. Another particularity of this case was its having a *BRAF* V600E mutation without an *EGFR* alteration (as 20%, 8/40 of all published cases), representing the second example of this discrepancy between genetic and epigenetic results (the first being reported as unilateral thalamic) [9]. Gliomas with concomitant mutations of H3K27M and *BRAF/FGFR1* are supposed to be associated with a better prognosis than other DMG, H3K27-altered according to some publications [7, 8]. As a result, it can be suggested that these molecular alterations (MAPK and H3K27M/EZHIP alterations) confer a different biological behavior, with a metastatic phenotype and/ or a slower local progression ultimately allowing the development of disseminated lesions. Arguing for this hypothesis, a previously published monothalamic tumor classified as ganglioglioma, H3K27M- and *BRAF* V600E-mutant presented secondary leptomeningeal dissemination 7 years after the initial diagnosis [10]. Further data is needed to understand this disseminated phenotype in detail.

In summary, we showed that despite the histopathological and molecular overlaps with DLGNT, DMG, H3K27-altered may be found to have, in exceptional cases, an initial disseminated radiological presentation.

## Supplementary Information

**Additional file 1. Table S1**: Summary of clinical data of cases from current series.

**Additional file 2. Table S2**: Summary of histopathological and molecular data of cases from current series.

**Additional file 3: Fig. S1**. Methylation-based t-SNE distribution. *t*-distributed stochastic neighbor embedding (t-SNE) analysis of DNA methylation profiles from the investigated tumors alongside selected reference samples. Reference DNA methylation classes: diffuse midline glioma H3 K27M mutant/EZHIP overexpressing (DMG_K27), diffuse midline glioma *EGFR*_altered (DMG_EGFR), glioblastoma, *IDH* wildtype, H3.3 G34 mutant (GBM_G34), pediatric glioblastoma, IDH wildtype, subclass *MYCN* (GBM_pedMYCN), glioblastoma, *IDH* wildtype, subclass RTK1 (GBM_RTK1), glioblastoma, *IDH* wildtype, subclass RTK2 (GBM_RTK2), pediatric glioblastoma, *IDH* wildtype, subclass RTK1 (GBM_pedRTK1), pediatric glioblastoma, IDH wildtype, subclass RTK2 (GBM_pedRTK2), glioblastoma, *IDH* wildtype, subclass mesenchymal (GBM_MES), diffuse leptomeningeal glioneuronal tumor, subtype 1 (DLGNT_1), and diffuse leptomeningeal glioneuronal tumor, subtype 2 (DLGNT_2).
