# Supplementary material for: Disseminated diffuse midline gliomas, H3K27-altered mimicking diffuse leptomeningeal glioneuronal tumors: a diagnostical challenge!
Source: Acta Neuropathol Commun. 2022 Aug 19;10:119. doi: 10.1186/s40478-022-01419-3 (PMC9392342; doi:10.1186/s40478-022-01419-3)
Supplement: Supplementary file 1 — Additional file 1. Table S1: Summary of clinical data of cases from current series. [file 40478_2022_1419_MOESM1_ESM.docx]

**Supplementary table 1. Summary of clinical data of cases from current series**

| **Case number** | **Clinico-radiological findings** | | | **Follow-up** | |
| --- | --- | --- | --- | --- | --- |
|  | **Age (yo)** | **Sex** | **Location** | **Treatment** | **Status (months)** |
| 1 | 14 | F | Right thalamus with LD | CT + RT | D, 15 |
| 2 | 13 | F | Right thalamus with LD | CT | A, 6* |
| 3 | 40 | F | LD and bone, without midline location | CT + RT | A, 4 |

A: Alive; CT: Chemotherapy; F: Female; LD: Leptomeningeal dissemination; RT: radiation therapy; yo: years-old.

*The radiological response by volumetric measurements was 80% reduction of lesions after treatment.
