# Supplementary material for: Disseminated diffuse midline gliomas, H3K27-altered mimicking diffuse leptomeningeal glioneuronal tumors: a diagnostical challenge!
Source: Acta Neuropathol Commun. 2022 Aug 19;10:119. doi: 10.1186/s40478-022-01419-3 (PMC9392342; doi:10.1186/s40478-022-01419-3)
Supplement: Supplementary file 2 — Additional file 2. Table S2: Summary of histopathological and molecular data of cases from current series. [file 40478_2022_1419_MOESM2_ESM.docx]

**Supplementary table 2. Summary of histopathological and molecular data of cases from current series**

| **Case number** | **Histopathological findings** | | | | **Immunohistochemical findings** | | | | | | | | | **Molecular findings** | | | |
| --- | --- | --- | --- | --- | --- | --- | --- | --- | --- | --- | --- | --- | --- | --- | --- | --- | --- |
|  | **Ca.** | **M (/5 HPF)** | **MVP** | **N** | **Olig2** | **ATRX** | **Syn.** | **NFP** | **MIB1 (%)** | **BRAFV600E** | **H3K27me3** | **H3K27M** | **EZHIP** | **1p del.** | **DNA-methylation (v12.5)** | **NGS** | **RNA seq.** |
| 1 | + | 1 | + | + | + | p. | +, no NI | + | 2 | - | Lost | - | + | + | **DMG H3K27M/EZHIP (0.99)**  DLGNT_1 (0.002)  DLGNT_2 (0.000) | *FGFR1* mut. *EGFR* WT* | - |
| 2 | - | 1 | - | - | + | p. | +, no NI | +++ | 5 | + | Lost | - | + | - | **DMG EGFR (0.95)**  DLGNT_1(0.000)  DLGNT_2 (0.000) | *BRAF* V600E, *EGFR* WT* | - |
| 3 | - | 21 | - | + | + | p. | - | + | 80 | - | Lost | + | - | - | **DMG H3K27M/EZHIP (0.82)**  DLGNT_2 (0.000)  DLGNT_1 (0.000) | *PIK3CA* mut., *H3F3A* K27M, *EGFR* WT* | - |

Ca.: calcifications; Del.: deletion; DLGNT_1: Diffuse leptomeningeal glioneuronal tumor, subtype 1; DLGNT_2: Diffuse leptomeningeal glioneuronal tumor, subtype 2; DMG: Diffuse midline glioma; HPF: high-power fields; M: mitoses; mut.: mutation; MVP: microvascular proliferation; N: necrosis; NA: not available; NFP : neurofilament protein; NGS: Next-generation sequencing; NI: neuropil island; p.: preserved; RNA-seq.: RNA-sequencing; Syn.: synaptophysin.

*including no amplification of *EGFR*.
