# Supplementary figures and images for: Disseminated diffuse midline gliomas, H3K27-altered mimicking diffuse leptomeningeal glioneuronal tumors: a diagnostical challenge!
Source: Acta Neuropathol Commun. 2022 Aug 19;10:119. doi: 10.1186/s40478-022-01419-3 (PMC9392342; doi:10.1186/s40478-022-01419-3)

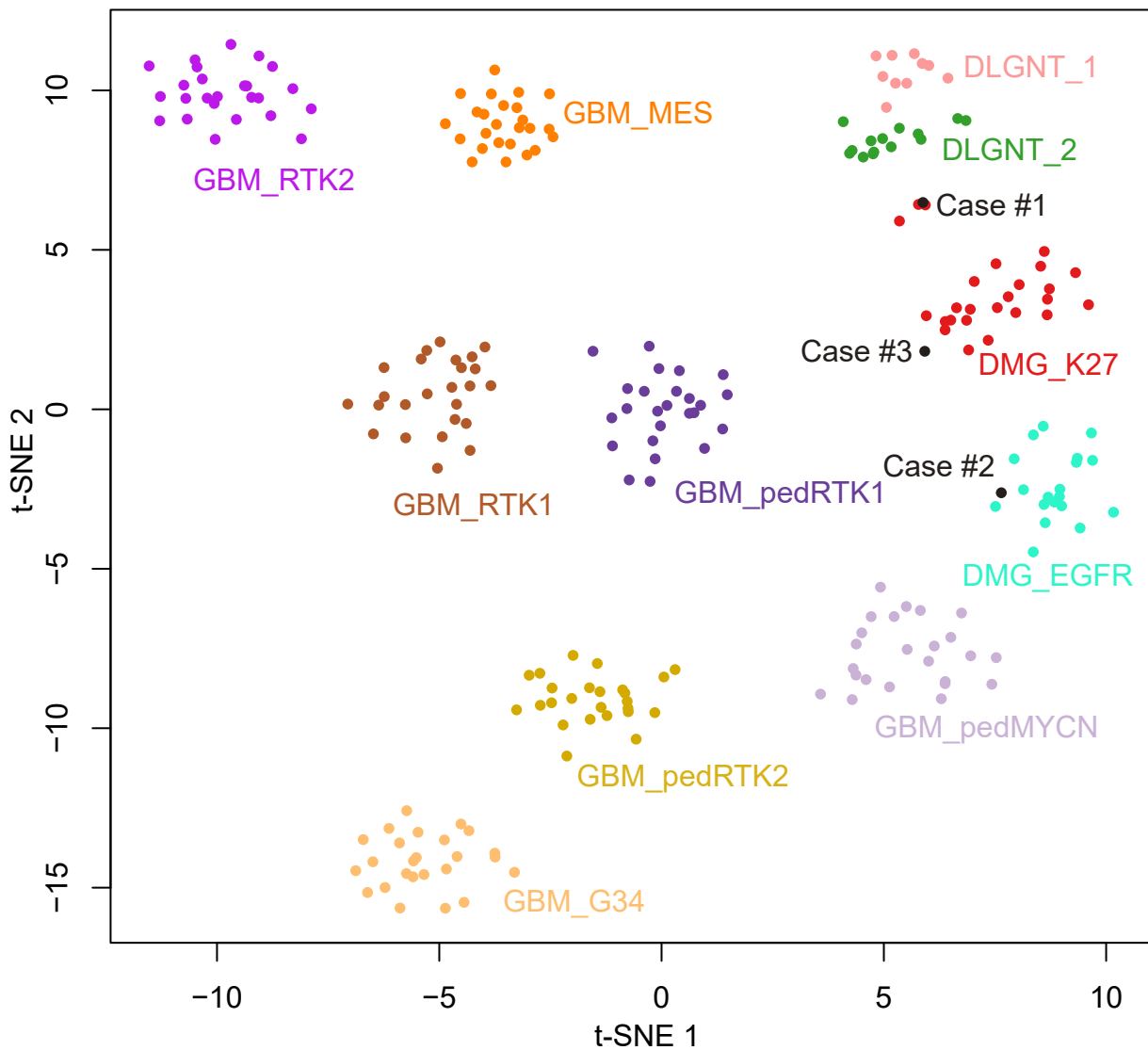

Supplement: Supplementary file 3 — Additional file 3: Fig. S1. Methylation-based t-SNE distribution. t-distributed stochastic neighbor embedding (t-SNE) analysis of DNA methylation profiles from the investigated tumors alongside selected reference samples. Reference DNA methylation classes: diffuse midline glioma H3 K27M mutant/EZHIP overexpressing (DMG_K27), diffuse midline glioma EGFR_altered (DMG_EGFR), glioblastoma, IDH wildtype, H3.3 G34 mutant (GBM_G34), pediatric glioblastoma, IDH wildtype, subclass MYCN (GBM_pedMYCN), glioblastoma, IDH wildtype, subclass RTK1 (GBM_RTK1), glioblastoma, IDH wildtype, subclass RTK2 (GBM_RTK2), pediatric glioblastoma, IDH wildtype, subclass RTK1 (GBM_pedRTK1), pediatric glioblastoma, IDH wildtype, subclass RTK2 (GBM_pedRTK2), glioblastoma, IDH wildtype, subclass mesenchymal (GBM_MES), diffuse leptomeningeal glioneuronal tumor, subtype 1 (DLGNT_1), and diffuse leptomeningeal glioneuronal tumor, subtype 2 (DLGNT_2). [file 40478_2022_1419_MOESM3_ESM.pdf]
